# Metalloproteinase meprin α regulates migration and invasion of human hepatocarcinoma cells and is a mediator of the oncoprotein Reptin

**DOI:** 10.18632/oncotarget.13975

**Published:** 2016-12-16

**Authors:** Osman Breig, Maïlyn Yates, Véronique Neaud, Gabrielle Couchy, Aude Grigoletto, Carlo Lucchesi, Johannes Prox, Jessica Zucman-Rossi, Christoph Becker-Pauly, Jean Rosenbaum

**Affiliations:** ^1^ University Bordeaux, INSERM, U1053, BordeAux Research in Translational Oncology, BaRITOn, Bordeaux, France; ^2^ Inserm, U1162, Génomique Fonctionnelle des Tumeurs Solides, Université Paris Diderot, Université Paris Descartes, Université Paris 13, Paris, France; ^3^ SIRIC BRIO, Bordeaux, France; ^4^ Unit for Degradomics of the Protease Web, University of Kiel, Germany

**Keywords:** RUVBL2, proteolysis, prognosis

## Abstract

Hepatocellular carcinoma is associated with a high rate of intra-hepatic invasion that carries a poor prognosis. Meprin alpha (Mep1A) is a secreted metalloproteinase with many substrates relevant to cancer invasion. We found that Mep1A was a target of Reptin, a protein that is oncogenic in HCC. We studied Mep1A regulation by Reptin, its role in HCC, and whether it mediates Reptin oncogenic effects.

MepA and Reptin expression was measured in human HCC by qRT-PCR and in cultured cells by PCR, western blot and enzymatic activity measurements. Cell growth was assessed by counting and MTS assay. Cell migration was measured in Boyden chambers and wound healing assays, and cell invasion in Boyden chambers.

Silencing Reptin decreased Mep1A expression and activity, without affecting meprin β. Mep1A, but not meprin β, was overexpressed in a series of 242 human HCC (2.04 fold, p < 0.0001), and a high expression correlated with a poor prognosis. Mep1A and Reptin expressions were positively correlated (*r* = 0.39, *p* < 0.0001). Silencing Mep1A had little effect on cell proliferation, but decreased cell migration and invasion of HuH7 and Hep3B cells. Conversely, overexpression of Mep1A or addition of recombinant Mep1A increased migration and invasion. Finally, overexpression of Mep1A restored a normal cell migration in cells where Reptin was depleted.

Mep1A is overexpressed in most HCC and induces HCC cell migration and invasion. Mep1A expression is regulated by Reptin, and Mep1A mediates Reptin-induced migration. Overall, we suggest that Mep1A may be a useful target in HCC.

## INTRODUCTION

Hepatocellular carcinoma (HCC) remains one of the deadliest cancers worldwide. The prognosis of HCC is dictated both by the status of the host liver, very often cirrhotic, and by the tumor extension. HCC is characterized by a very frequent intra-hepatic invasion, notably of portal veins, and by microvascular invasion, which carries a very poor prognosis [1]. Although a number of mechanisms responsible for HCC cells migration and invasion have been identified, this knowledge has not so far impacted the handling of patients. There is thus a great need for discovering new actionable targets.

In the last few years, we have discovered the overexpression in HCC of Reptin, or RUVBL2, a member of the AAA+ ATPase family [2]. Reptin, and its homolog and partner protein, Pontin/RUVBL1 (also found overexpressed in HCC [3]) are part of several protein complexes that have pleiotropic functions (for review, see [4]). Briefly, they are involved in the remodeling of chromatin, the regulation of gene transcription through various mechanisms, and they also act as chaperones for proteins of the Phosphatidylinositol Kinase-like Kinases family such as mTOR, ATM or DNA-PKcs. We found that both proteins were required for the growth and viability of HCC cells [2, 3, 5] and that silencing Reptin within established HCC xenografts induced tumor regression, thus establishing Reptin as a potential target in HCC [6]. There is also evidence showing that Reptin silencing reduces invasion of cancer cells from kidney [7] and prostate [8]. The downstream mediators of Reptin underlying its oncogenic effects remain however to be found.

Meprin α is a secreted metalloproteinase that can cleave a potentially large number of protein substrates, many of them being highly relevant for cancer [9]. Those include extracellular matrix and related proteins (pro-collagen I, fibronectin, SPARC, osteopontin), cytokines (pro-IL1β, IL6), growth factors (VEGF-A, CTGF), membrane proteins (occludin), and other proteinases (MMP1, ADAM10) [10]. Meprin α had so far received little attention in cancer, with limited evidence showing that it could stimulate cancer cell proliferation [11], migration [12] and invasion [13]. While this work was in progress, however, OuYang et al. showed that it could induce HCC cell migration and invasion [14]. Interestingly, there are already available good meprin α inhibitors like actinonin [15] that can be used *in vivo* [16–18], and more specific inhibitors are currently under development [19], raising the hope that meprin α may become a clinically useful target.

Knowing that Reptin regulates the expression of many genes [20, 21], we hypothesized that it might regulate meprin α. Thus, in this study, we have investigated the regulation of the expression of meprin α by Reptin in HCC, the effects of meprin α on HCC cell phenotype, and whether meprin α mediated the oncogenic effects of Reptin.

## RESULTS

### Reptin silencing decreases the expression of meprin α

**Figure 1:**
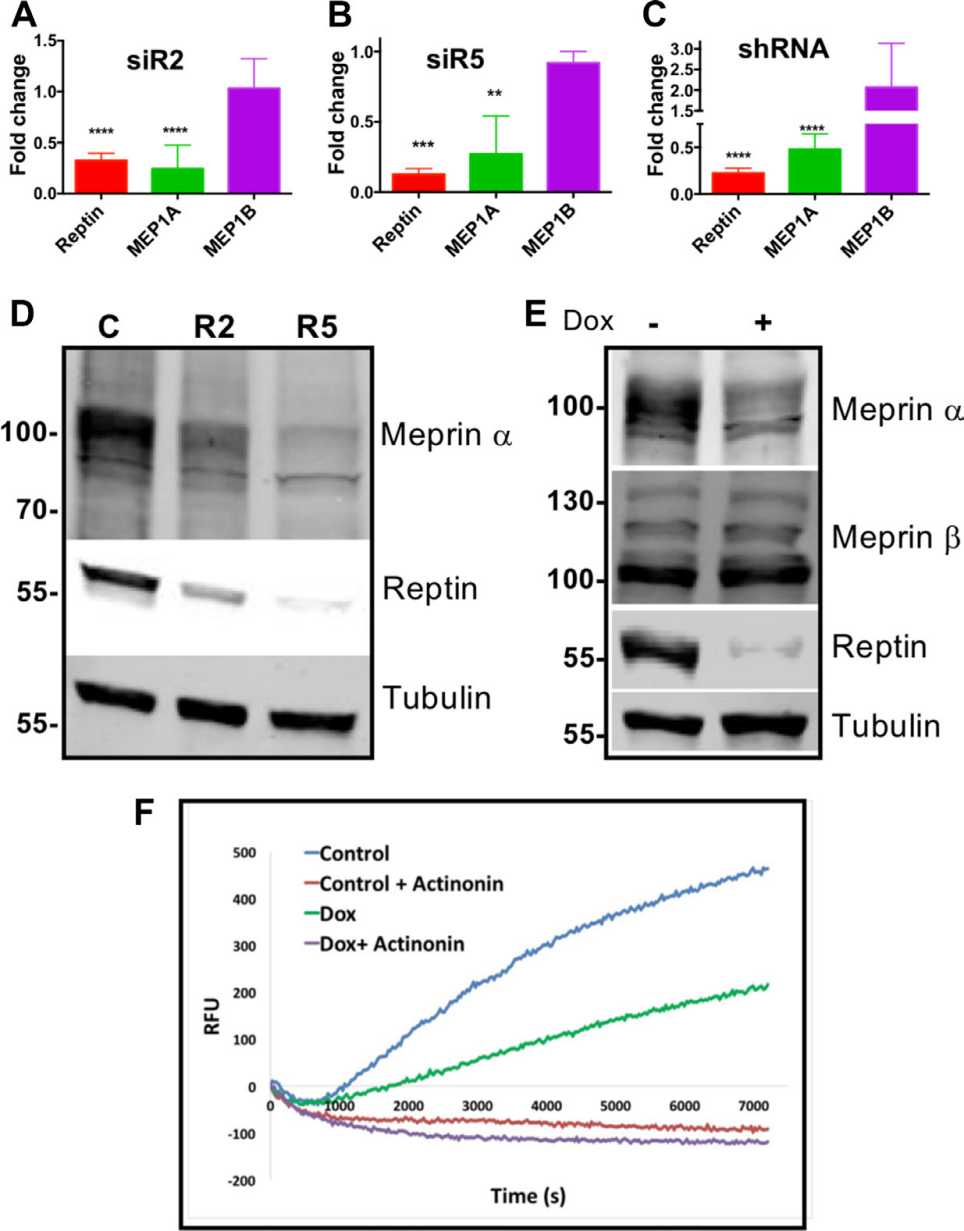
Reptin regulates expression of meprin a (**A**) Expression of Reptin, meprin a and meprin α mRNA was examined by qRT-PCR in HuH7 cells following Reptin silencing with the siR2 siRNA. Results are expressed as fold changes as compared to treatment with a control siRNA (*p* < 0.0001; *n* = 8 for Reptin and MEP1A, *n* = 2 for MEP1B). (**B**) Same experiment with the siR5 siRNA (*p* = 0.0004; *n* = 3 for Reptin and MEP1A, *n* = 2 for MEP1B). (**C**) Same experiment but Reptin was silenced with a doxycycline-inducible shRNA. Results are expressed as fold changes as compared to non-induced cells (*p* < 0.0001; *n* = 3 for Reptin, *n* = 9 for MEP1A, and *n* = 4 for MEP1B). (**D**) Western blot of HuH7 extracts showing decreased expression of meprin α following silencing Reptin with the R2 or R5 siRNAs, as compared to control cells. Antibody specificity was demonstrated in silencing experiments (see Figure 3C). Tubulin is shown as a loading control. (**E**) Similar experiment but Reptin was silenced with the inducible shRNA. Meprin β expression was not altered. (**F**) Reptin silencing decreased meprin α proteolytic activity in HuH7 conditioned medium. Reptin was silenced using the inducible shRNA by adding doxycyxline (Dox). The meprin a inhibitor Actinonin was used as a specificity control.

Reptin expression was efficiently silenced in HuH7 cells using two different siRNAs, or with a doxycycline-inducible shRNA, as described previously [2, 6]. This resulted in a significantly decreased expression of meprin α mRNA, whereas the expression of meprin β was not decreased (Figure 1A–1C). Similar results were obtained in another HCC cell line (Hep 3B (Supplementary Figure S1A). Furthermore, meprin α, but not meprin β protein expression was also reduced as shown by Western blot (Figures 1D–1E, Supplementary Figure S1D). Finally, meprin α proteolytic activity in the conditioned medium was decreased upon Reptin silencing. As a control, we confirmed that the substrate cleaving activity was abrogated by the meprin inhibitor actinonin (Figure 1F).

### Meprin α is overexpressed in human HCC and its expression correlates with that of Reptin

**Figure 2:**
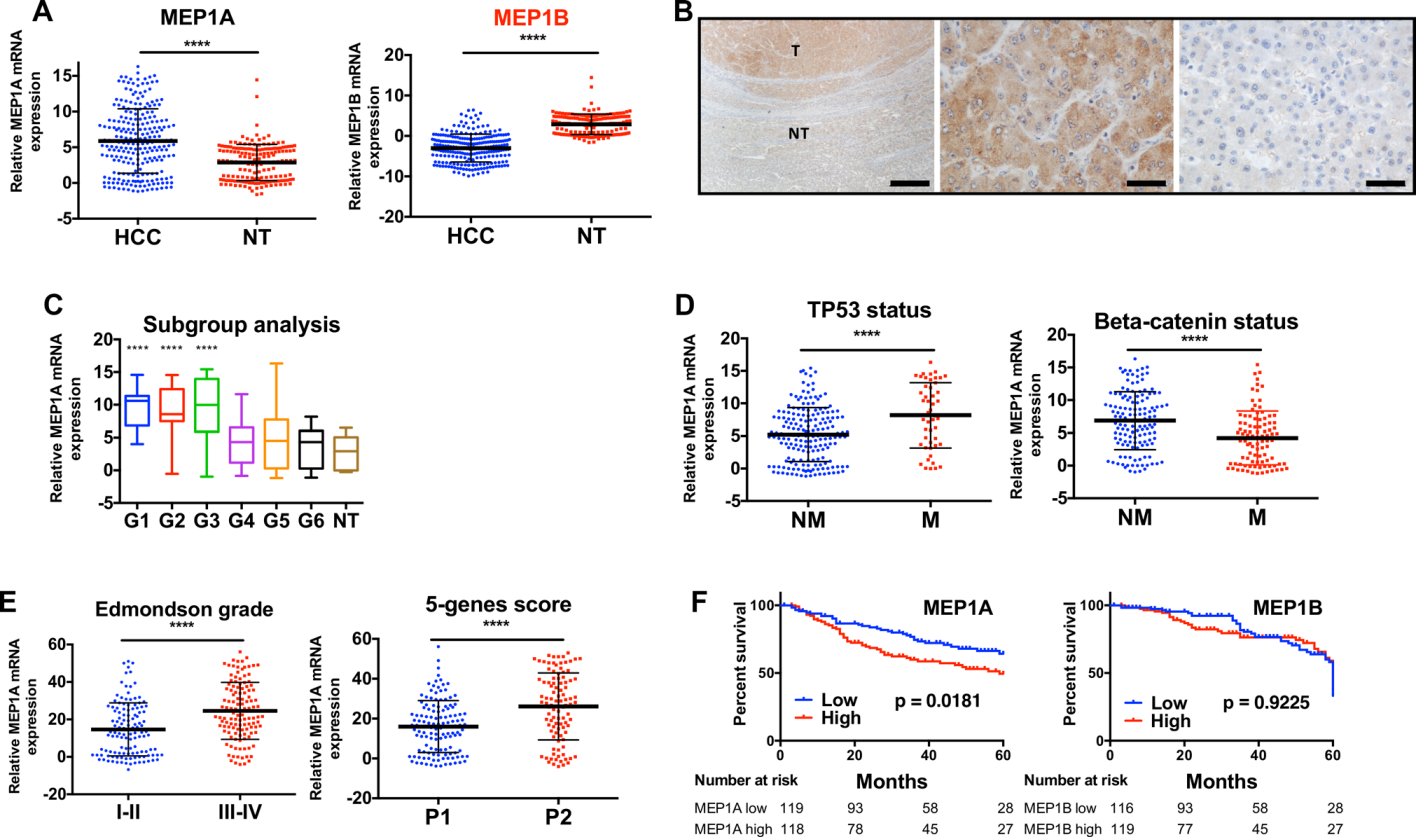
Meprin a is overexpressed in human HCC and its expression correlates with that of Reptin (**A**) Meprin α (left) and meprin β (right) expression was examined by qRT-PCR in 242 HCC and 166 non-tumor (NT) livers. Gene expression was normalized with the RNA ribosomal 18 S (*p* < 0.0001). (**B**) Analysis of meprin α expression by immunohistochemistry at the border between tumor (T) and non-tumor (NT) liver in a patient with a HCC (left). The middle picture is a close-up view of the tumor and the right one a close-up view of the non-tumor part. The bar corresponds to 500 μm on the left panel and to 50 μm on the other ones. (**C**) Box and whisker plot showing the expression of meprin α mRNA according to the transcriptomic classification of HCC [29] and in non-tumor liver (**** indicates *p* < 0.0001 as compared to non-tumor liver; other comparisons did not yield significant results). (**D**) Meprin α expression according to the gene mutation status of the tumors. Left, TP53 (*p* < 0.0001), right, beta-catenin (*p* < 0.0001) (Non mutated, NM; mutated, M). (**E**) Meprin α expression according to the Edmondson grade of tumors (left, *p* < 0.0001) or to the 5-genes prognosis score, P1 patients having a better prognosis [28] (right, *p* < 0.0001). (**F**) Kaplan Meier analysis demonstrating that patients with high meprin α expression (left) have a significantly poorer prognosis than patients with a low expression (logrank test). A similar analysis for meprin β (right) does not demonstrate any prognosis value.

Expression of meprin α, meprin β and Reptin mRNAs was assessed using RT-PCR on a previously described series of 242 HCC from the French Biological Resource Center on HCC [22]. Meprin α mRNA level was significantly higher in HCC samples as compared to non-tumor liver (2.04 fold, *p* < 0.0001; Figure 2A). On the other hand, meprin β expression was not increased, and was even significantly decreased (*p* < 0.0001) when comparing HCC to non-tumor liver (Figure 2A).

Immunohistochemistry experiments in 3 HCC tumors confirmed in all cases an overexpression of Meprin α in HCC as compared to the non-tumor liver in the same patient. Furthermore, these experiments demonstrated unambiguously that Meprin α overexpression takes place in tumor cells and not stromal cells (Figure 2B).

Analysis of HCC subgroups revealed that meprin α expression was especially high in transcriptomic groups G1, G2 and G3 (Figure 2C). These groups are characterized by a higher rate of cell proliferation, and accordingly meprin α expression was significantly correlated with that of Ki67 mRNA (Spearman *r* = 0.41, *p* < 0.0001). Groups G1-G3 are also enriched in patients with TP53 mutations [23] and we found indeed that meprin α expression was significantly higher in patients with TP53 mutation, and conversely lower in those with CTNNB1 mutations, that are enriched in G4-G6 patients (Figure 2D). There was no significant difference in meprin α expression whether the cause of liver disease was excess alcohol consumption, B or C viral hepatitis, hemochromatosis or metabolic liver disease (not shown).

Additionally, several data pointed to a significant correlation of high meprin α expression with a poor prognosis. First, meprin α expression was significantly higher in tumors from Edmonson grades II-IV, as compared to grades I-II (Figure 2E). We previously found that a score based on the expression of the 5 genes *HN1, RAN, RAMP3, KRT19*, and *TAF9*, was associated with the survival of HCC patients following surgery [22]. We found here that meprin α expression was significantly higher in tumors from patients with high 5-gene prognosis score (Figure 2E). Finally, we performed a Kaplan-Meier analysis by first dichotomizing the patients as meprin low or high according to the median of expression in all samples. This analysis showed that the overall survival of patients was significantly worse in patients with a high expression of meprin α, whereas meprin β expression carried no prognostic value (Figure 2F).

Very interestingly, and in line with our results showing the regulation of meprin α expression by Reptin, we found that meprin α expression was significantly and positively correlated with that of Reptin in HCC (Spearman *r* = 0.23, *p* = 0.0003), whereas no correlation was found for meprin β (Spearman *r* = 0.083, *p* = 0.20).

### Generation of tools for loss and gain of function of meprin α

**Figure 3:**
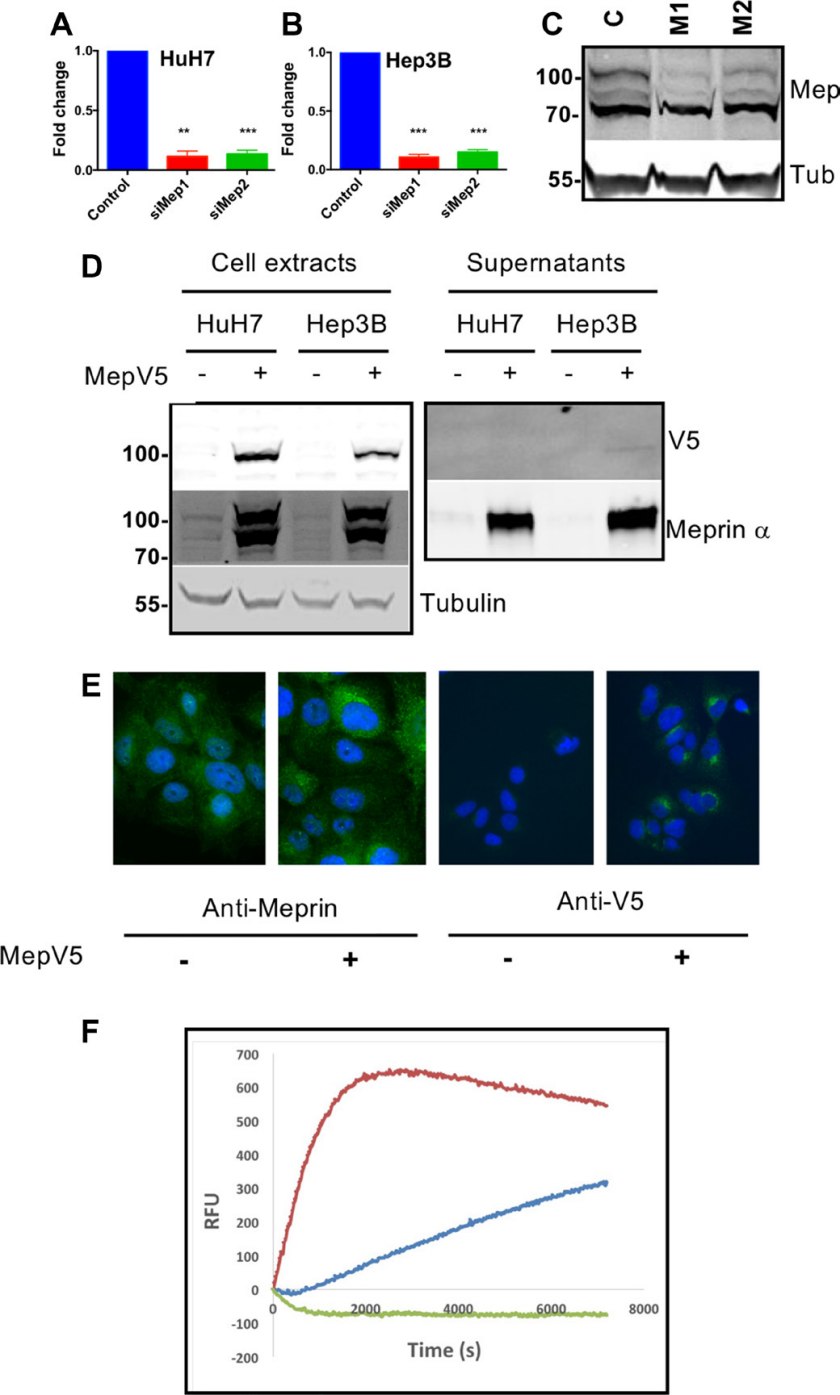
Generation of tools for down- or upregulation of meprin a expression (**A**) Meprin α expression was analyzed by qRT-PCR in HuH7 cells following transfection of M1 or M2 meprin a siRNAs (*p* = 0.0002, *n* =3). (**B**) Same experiment in Hep3B cells (*p* = 0.0002, *n* =3). (**C**) Western blot of HuH7 extracts showing decreased expression of meprin α following transfection with a control siRNA (C) or with the M1 or M2 siRNAs. (**D**) Overexpression of meprin α with a V5 tag in HuH7 or Hep3B cells. Stable cell lines were established as described in Methods. (**E**) Immunofluorescence for Meprin (left) or the V5 tag (right). (**F**) Meprin α proteolytic activity in the conditioned medium is expectedly increased in HuH7 cells overexpressing meprin-V5.

For loss of function, we designed two siRNAs targeting meprin α. Both siRNAs efficiently depleted meprin α mRNA and protein (Figure 3A–3C). For gain of function experiments, we used two complementary strategies. First, we constructed HuH7 and Hep3B cell lines constitutively overexpressing meprin α with a V5 C-terminal tag. Because the C-terminus of meprin α is removed during the maturation and secretion process, the tag is no more present in the secreted protein and does not interfere with the proteolytic activity. As shown on Figure 3D, the V5-tagged protein was found in large amounts in cell extracts where it can be detected with both a meprin α and a V5 antibody. On the other hand, the overexpressed protein is detectable only with the meprin α antibody in the supernatant of the cells. Immunofluorescence with both anti-meprin α and anti-V5 antibodies confirm the overexpression (Figure 3E). Finally, the supernatant of meprin α-overexpressing cells contains a high level of proteolytic activity (Figure 3F), demonstrating the functionality of the engineered protein. In other experiments, cells were treated by addition of recombinant human meprin α in the culture medium.

### Meprin α has a limited effect on HCC cell proliferation

**Figure 4:**
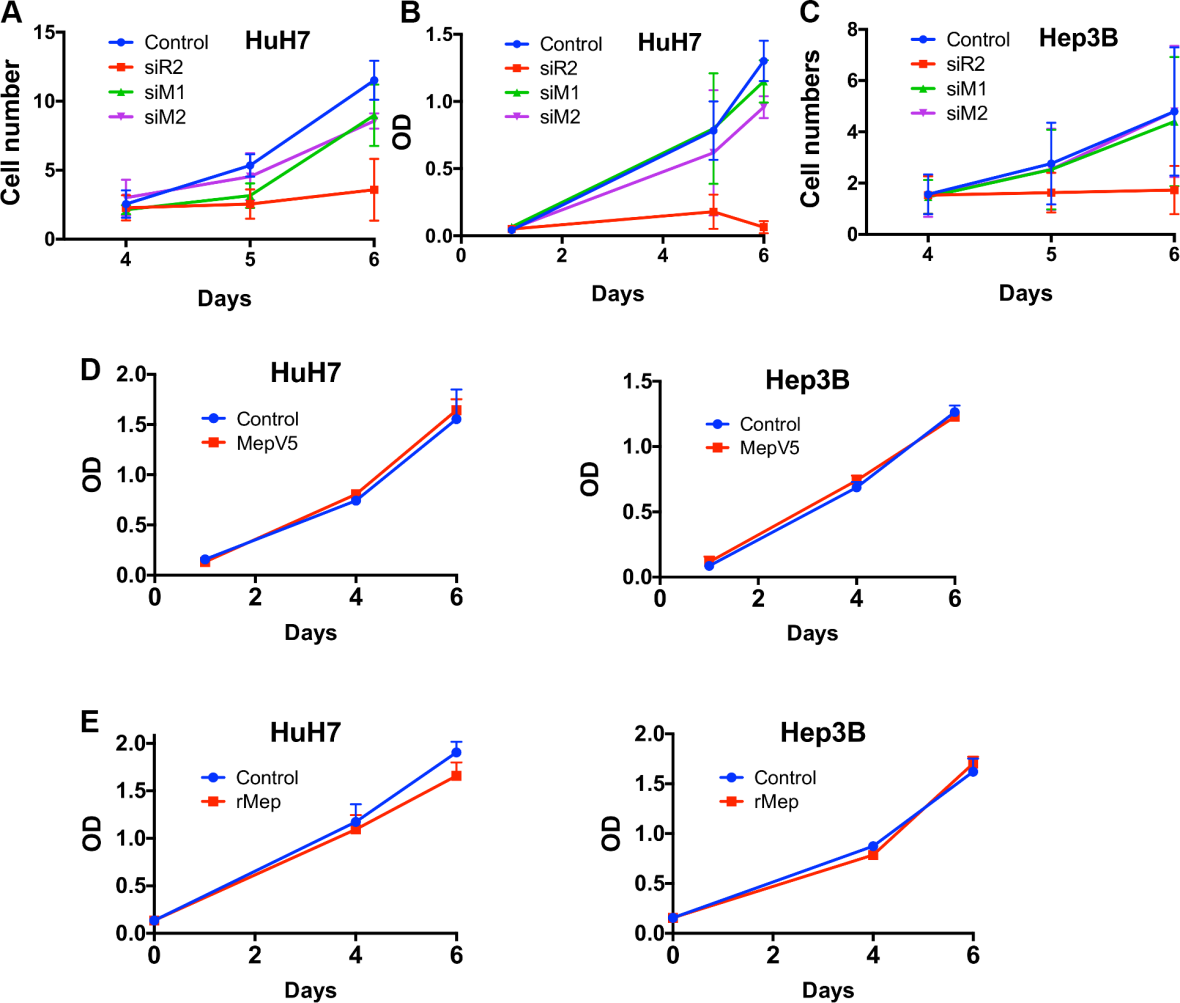
Meprin a has limited effect on cell proliferation (**A**) HuH7 cell proliferation was assessed by direct cell counting in cells transfected with a control siRNA, or siRNAs targeting Reptin or meprin α (M1 and M2). Reptin silencing significantly decreased proliferation. The M2 siRNA had no significant effect whereas the M1 siRNA has a slight but significant effect (*p* = 0.05, *n* = 3). (**B**) MTS assay in HuH7 cells showing no significant effect of the two meprin siRNAs on proliferation. (**C**) Cell counting experiment in Hep3B cells showing that Reptin, but not meprin silencing, reduces proliferation (*n* = 3). (**D**) Growth of HuH7 (left) or Hep3B cells (right) overexpressing meprin-V5 assessed with a MTS assay (*n* = 3). (**E**) Growth of HuH7 (left) or Hep3B cells (right) treated with 20 nM recombinant meprin α assessed with a MTS assay (*n* = 3).

Since there was some evidence suggesting that meprin α could induce cell proliferation [11, 14], we investigated this issue in two HCC cell lines. Meprin α was silenced with siRNAs and cell proliferation was followed over 6 days. As a positive control, Reptin silencing expectedly greatly decreased cell growth of both HuH7 and Hep3B cells (Figure 4A–4C). Meprin α silencing on the other hand had only limited effects: one of the two siRNAs (M1) significantly decreased cell growth in Huh7 as shown by direct counting, although the effect was minimal and was not found with the MTS assay in the same cell line, nor by direct cell counting in Hep3B cells. Similarly, the growth of HuH7 and Hep3B cells stably overexpressing V5-tagged meprin α (Figure 4D), or treated with recombinant meprin α (Figure 4E) was identical to that of control cells.

### Meprin α regulates HCC cells migration and invasion

**Figure 5:**
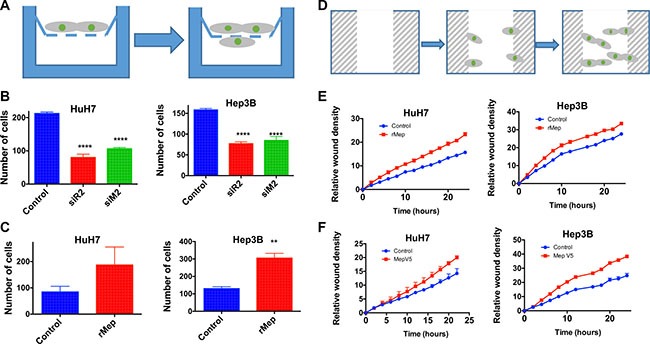
Reptin and meprin α regulate migration of HCC cells (**A**) Experimental set-up for the migration assay in Boyden chambers. Migrating cells are counted on the bottom of the filter. (**B**) Migration of HuH7 cells (left panel) or Hep3B (right) was assessed in Boyden chambers following silencing of Reptin (siR2) or meprin a (siM2) (*p* < 0.0001, *n* = 3) (**C**) Boyden chamber migration assay after addition of recombinant human meprin α (rMep) to HuH7 (left) or Hep3B cells (right) (*n* = 3; HuH7, *p* = 0.06, Hep3B, *p* = 0.004). (**D**) Experimental set-up for the Incucyte assay. Following a wound in the monolayer, cells migrate and repopulate the wound. The migration is assessed using time-lapse microscopy. (**E**). Migration of HuH7 (left) or Hep3B cells (right) with or without added recombinant meprin α was assessed using the Incucyte wound healing assay. (*n* = 3; two-way ANOVA, *p* = 0.05 for both). (**F**) Same experiment but using cells overexpressing meprin-V5 or control cells (*n* = 4; two-way ANOVA, *p* = 0.05 for both).

Cell migration was first studied using a Boyden chamber assay (Figure 5A). Meprin α silencing with siRNA led to a decreased migration in both HuH7 and Hep3B cells, which was in a similar range as obtained following silencing of Reptin (Figure 5B). Conversely, addition of recombinant meprin α to the medium enhanced cell migration in this assay (Figure 5C). We used an independent test where migration was assessed following wounding of a monolayer, using live cell imaging over 24 hours (Figure 5D). This assay confirmed that addition of recombinant meprin α in the culture medium increased migration (Figure 5E). In addition, we also show that HuH7 or Hep3B cells stably overexpressing V5-tagged meprin α demonstrate an increased ability to migrate (Figure 5F).

**Figure 6:**
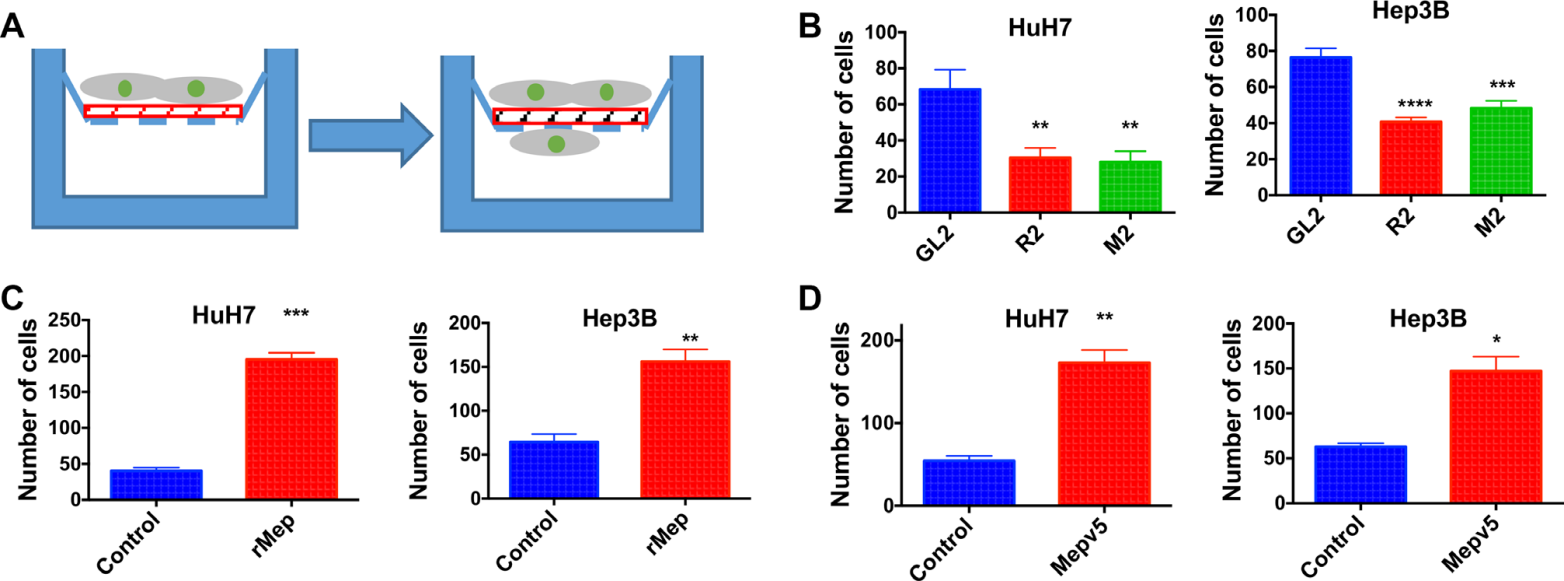
Reptin and meprin α regulate invasion of HCC cells (**A**) Experimental set-up for the invasion assay. The design is the same as in (A) except that a Matrigel layer (red) was added. (**B**) Invasion across Matrigel was measured in Boyden chambers for HuH7 (left) or Hep3B cells (right) following silencing of Reptin or meprin α (*n* = 3; HuH7, *p* = 0.01, Hep3B, *p* = 0.005). (**C**) Invasion assay after addition of recombinant human meprin α (rMep) to HuH7 (left) or Hep3B cells (right) (*n* = 3; HuH7, *p* = 0.0003, Hep3B, *p* < 0.0001). (**D**) Invasion assay using HuH7 (left) or Hep3B cells (right) overexpressing meprin-V5, as compared to control cells (*n* = 3; HuH7, *p* = 0.002, Hep3B, *p* < 0.01).

Finally, we also assessed the invasion capacity of HCC cells using Matrigel-coated Boyden chambers (Figure 6A). Here also, in both HuH7 and Hep3B cells, meprin α silencing decreased invasion (Figure 6B) whereas addition of recombinant meprin α or overexpression of V5-tagged meprin α stimulated it (Figure 6C–6D).

Since changes in migration and invasion could occur because of changes in the adhesive properties of the cells, we assessed adhesion of control and meprin-V5 overexpressing HuH7 cells on serum-coated wells and found no significant differences in adhesion between the two cell populations (Supplementary Figure S2).

### Meprin α as a mediator of Reptin

**Figure 7:**
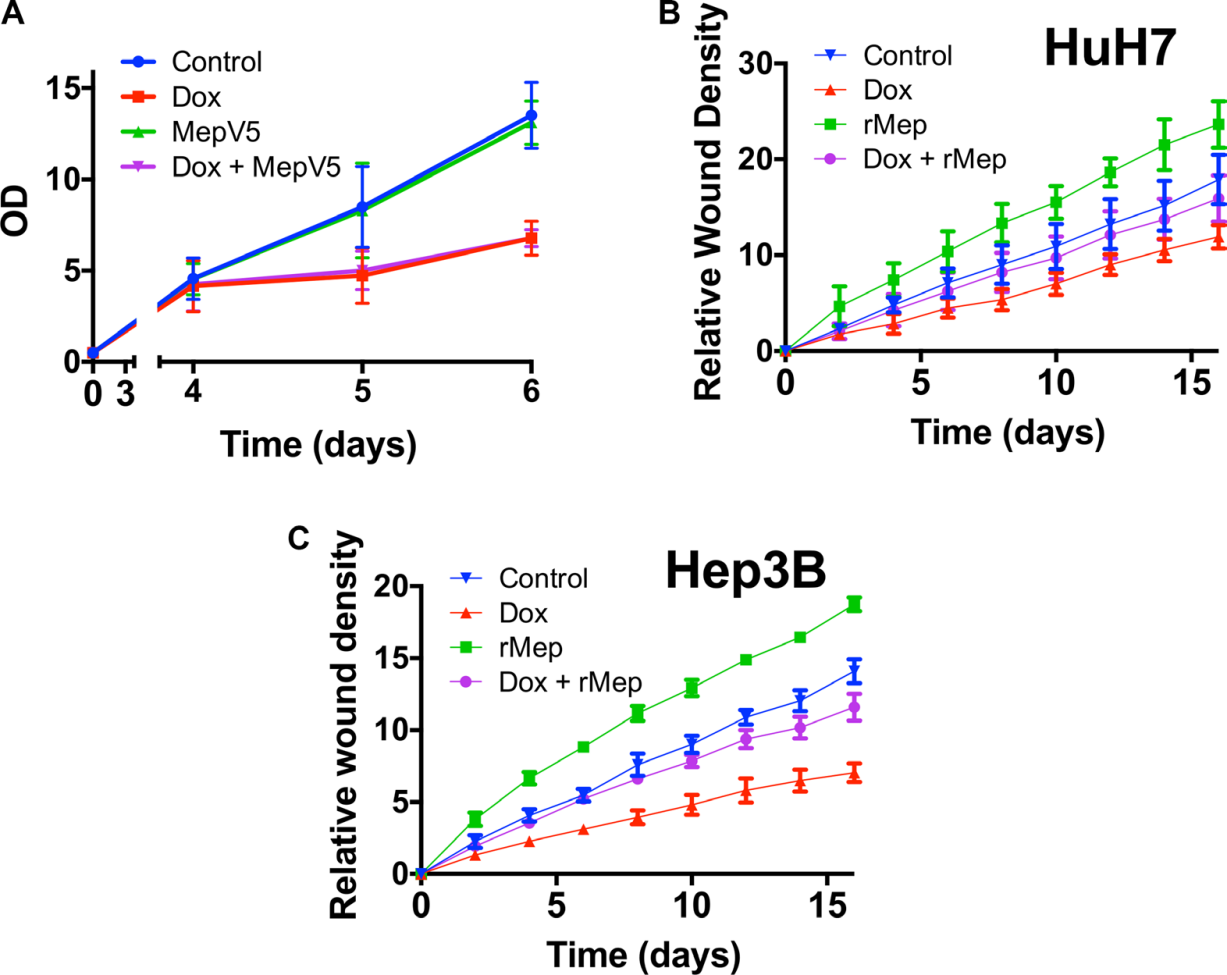
Meprin α is a mediator of Reptin for cell migration (**A**) MTS assay with HuH7 cells harboring an inducible Reptin shRNA without Doxycycline (Control), with Doxycycline (Dox), and the same cells also overexpressing meprin V5 with or without Doxycycline. (**B**) Migration of HuH7 cells harboring an inducible Reptin shRNA was assessed using the Incucyte wound healing assay in the above mentioned conditions (rMep = with added 20 nM recombinant meprin α; the graph shows the mean of 3 independent experiments) (**C**) Same as in (B) but with Hep3B cells (*n* = 3). In both (B) and (C), differences between graphs were significant by 2-way ANOVA (*p* = 0.05). Silencing Reptin significantly decreased migration from 2 h onwards. Similarly, overexpressing meprin whether Reptin is silenced or not, significantly increased migration from 2 h onwards. On the other hand, there was no difference between control cells and cells with Reptin silencing plus added recombinant meprin until 12 h (HuH7) or 6 h (Hep3B).

Because Reptin silencing strongly regulates meprin α expression, we asked whether meprin α down-regulation in this setting was responsible for some of the effects brought by Reptin silencing. To address this question, we used cells where Reptin was silenced with an inducible shRNA, leading to meprin α down-regulation, and we restored meprin α activity either by using cells expressing V5-tagged meprin α or application of the recombinant protease. We first tested whether this strategy would reverse the anti-proliferative effect imposed by Reptin silencing. Here we used cells expressing both the inducible Reptin shRNA and V5-tagged meprin α. We found that as expected, induction of the Reptin shRNA slowed cell growth, but this was not different in cells co-expressing V5-tagged meprin α (Figure 7A). We verified that Reptin silencing in these cells did not detectably alter the expression of V5-tagged meprin α (not shown).

We then turned to cell migration. Previous publications have shown that Reptin silencing reduced the migration of prostate [8] and kidney [7] cancer cells. We found similarly that Reptin silencing in HuH7 cells strongly reduced cell migration as assessed both by a wound healing (Figure 7B–7C) and a Boyden chamber (Figure 5B) assay. Interestingly, overexpression of V5-tagged meprin α was able to restore a close to normal migration in cells where Reptin was silenced (Figure 7B–7C).

## DISCUSSION

Here, we found that Reptin strongly regulated the expression of the secreted metalloprotease meprin α. As outlined in the Introduction, meprin α is a broad-spectrum extracellular proteinase with a high potential relevance to cancer. This stems from the seminal studies by Lottaz et al. who have shown that meprin α was overexpressed in most cases of a small series of colon cancer and was present in active form in the tumors [24]. The same researchers found that meprin α enhanced the migration of colon cancer cells and promoted angiogenesis [12].

We found that meprin α was consistently overexpressed in a large series of human HCC. More importantly, we found that overexpression of meprin α was correlated with several indexes of poor prognosis. It was indeed significantly overexpressed in patients with poorly differentiated tumors and in those classified as P2 using the 5-genes prognosis score that we defined previously [22]. Finally, mean survival following surgery was significantly shorter in patients with high meprin α levels as compared to those with lower levels. These results are in agreement with those recently reported in an Asian series [14] altogether suggesting the robustness of meprin α as a prognosis factor in HCC. Moreover, our study demonstrated that only meprin α, but not the highly related meprin β, was overexpressed in HCC. There was even a significant down-regulation of meprin β in HCC although the level of meprin ß expression was not associated with prognosis of patients. Meprin α shares a large homology with meprin β. A major difference resides in the presence of an inserted domain in meprin α that is cleaved by furin in the Golgi compartment, leading to the secretion of meprin α. Meprin β instead remains membrane bound, where it acts as an ectodomain sheddase cleaving type I transmembrane proteins [25]. Although both meprins share a number of substrates as shown notably by us [10], some are specific to one or the other, which may impact on their role in cancer. For instance, only meprin β but not meprin α can cleave osteopontin [26], a protein endowed with pro-metastatic properties notably in HCC [27, 28]. Conversely, only meprin α can cleave the tight junction protein occludin [29], which could favor cell migration.

In our hands, meprin α had little effect on cell proliferation. These results are in contrast with those recently reported in HCC by another group [14]. There is no ready explanation for the discrepancy between the two studies. It must be pointed out that we obtained concordant results with both loss and gain of function of meprin α, the latter with two modalities (overexpression and use of recombinant meprin α) in two different cell lines. A technical issue is unlikely since the same procedures greatly affected HCC cell migration in our hands. Besides HCC, there is only limited evidence for a role of meprin α in cell proliferation. A single study has shown that recombinant meprin α could stimulate colorectal cancer cell proliferation via the transactivation of the EGF receptor [11]. In addition, others have shown that the meprin inhibitor actinonin reduced the growth of a large number of tumor cell lines, a finding that we also observed with HCC cells (data not shown). However, actinonin is also a potent inhibitor of human mitochondrial peptide deformylase, which likely explains most of its anti-proliferative effect [30]. Thus, we conclude that meprin α has no major involvement in the proliferation of HCC cells.

On the other hand, our data agree very well with those of OuYang et al. [14] on the effects of meprin α on cell migration and invasion. Indeed, cells stably overexpressing meprin α, or treated with addition of recombinant meprin α exhibited a higher rate of migration and invasion. Conversely, silencing meprin α resulted in decreased migration and invasion. These data are also reminiscent of published results with MDCK [12] and Caco-2 [11] cells. Altogether, we conclude that meprin α is a potent inducer of HCC cell migration and invasion. The mechanisms used by meprin α to regulate migration and invasion remain to be determined. They are likely complex since meprin α has a large number of substrates including extracellular matrix proteins, cytokines, growth factor receptor ligands, all of them being potentially able to influence migration and invasion behavior.

A very original finding of our study is the demonstration of meprin α being a mediator explaining the effects of Reptin on cell migration. Reptin has been consistently shown overexpressed in human HCC [2, 31–33] where a high level of expression is correlated with a poor prognosis [2]. We and others have shown that Reptin is a potent mediator of oncogenesis and is considered as an attractive target in cancer [2, 3, 5–7, 34]. However, because Reptin has pleiotropic effects, its direct targeting may prove difficult and could result in untoward effects. Thus, the search for Reptin downstream targets responsible for its oncogenic effects is warranted. Here we have shown that Reptin is a strong regulator of meprin α expression in tumor hepatocytes since silencing Reptin resulted in a robust reduction in meprin α expression at mRNA, protein and activity levels. Very interestingly, Reptin and meprin α mRNA expression levels are positively correlated in a large series of human HCC samples, suggesting that Reptin may indeed be involved in regulating meprin α expression within these tumors. This appears rather specific for HCC since a correlation analysis conducted on public data from the TGCA showed no such correlation for 574 breast or 270 colon cancer samples (not shown). On the other hand, Reptin did not regulate meprin β expression, and there was no correlation between the expression of these two genes in human HCC. A transcriptional regulation of meprin α by Reptin is likely since both meprin α mRNA and protein are regulated upon Reptin silencing. However, since Reptin is mostly a repressor of transcription [35], it is possible that it acts by down-regulating a repressor of meprin α expression. More importantly, we found that restoring the expression of meprin α in cells where Reptin was silenced also restored a normal migration ability to the cells. Although there have been some reports showing that Reptin could regulate tumor cell migration [7, 8], the responsible mechanisms have not been evidenced. Based on our findings, we thus propose that Reptin regulates tumor cell migration and invasion through its ability to control the expression of meprin α.

In conclusion, our data confirm that the metalloproteinase meprin α is overexpressed in human HCC where a high expression correlates with a poor prognosis. Meprin α is a strong inducer of HCC cells migration and invasion. Furthermore, we demonstrate that meprin α expression is regulated by the oncoprotein Reptin, and that it mediates at least part of the effect of Reptin on HCC cell migration. Being secreted in the extracellular medium, meprin α may be a relevant target to block HCC progression.

## MATERIALS AND METHODS

### Cell culture

The human hepatocellular carcinoma cell lines HuH7 [36] and Hep3B [37] were grown in Dulbecco's modified Eagle's medium (DMEM) supplemented with 10% fetal calf serum in a 5% CO_2_ atmosphere at 37°C. Cells were authenticated using short tandem repeat analysis and tested for mycoplasma contamination on a regular basis.

### Transfection and siRNAs

For silencing meprin α, we used two different siRNAs, siM1 and siM2 (Table 1). For Reptin, we used the previously described siR2 [2, 6]. Transfections were carried out as described [5]. In some experiments, Reptin was silenced in cells harboring an integrated shRNA inducible with doxycycline [6].

**Table 1:** siRNA sequences

siM1	5'-GGUUACCAGCACAACUUUGTT-3'
siM2	5'-GACUGUAAUUGUUUUAGAATT-3'

### Construction of cell lines overexpressing meprin α

A lentiviral vector coding meprin α with a C-terminal V5 tag was obtained from Thermo Scientific [38]. HuH7 and Hep3B cells were transduced and stable cell lines were obtained using selection with blasticidin. As a control, cells were transduced with lentiviral particles containing the empty pLX304 plasmid.

### Purification of recombinant meprin α and meprin activity assay

Human meprin α was produced and purified using a baculovirus expression system as described [19]. Meprin α activity was assayed using a synthetic peptide [(MCA)-YVADAPK-(K-e-DNP)] as described [39]. In brief, cells were lysed and 100 μg of total protein was analyzed for cell lysate fractions using 20 mM Hepes buffer for dilution in a final volume of 100 μl. For inhibition of meprin α activity, samples were treated with 10 μM actinonin for 15 min at RT prior to the cleavage assay. Cell culture supernatants were treated with 10 μg/ml trypsin for 30 min at 37°C to activate the secreted proform of meprin α and afterwards ovomucoid was added to the solution to inhibit completely the trypsin activity. The activity assay was performed as described above using 100 μl of undiluted cell culture supernatant.

### Migration and invasion assays

Cell migration was assessed using two methods. First, using a Transwell assay, 5 × 10^4^ cells were seeded in the upper chamber of a 24 w Transwell plate. After 24 hours, cells that migrated to the bottom chamber were fixed in 3% paraformaldehyde, stained with Hoechst and counted. In other experiments, migration was assessed with a wounding assay, using the Incucyte system (Essen Bioscience).

Cell invasion was assessed using a Boyden chamber assay with Matrigel-coated wells, as described [40].

### Cell growth assay

Cell proliferation assays were performed using tetrazolium compound based CellTiter 96^®^ A^Queous^ One Solution Cell Proliferation (MTS) assay (Promega) as previous described [5].

### qRT-PCR

For human tissue samples, RNA was isolated using the Maxwell Tissue LEV Total RNA Purification kit and instrument (Promega); 1 μg of RNA was reverse transcribed using MultiScribe reverse transcriptase and random hexamers (Applied Biosystems). Quantitative RT-PCR was performed using predesigned TaqMan probes (Hs00194410_m1, Hs00195535_m1, and -Hs00272632_m1 for MEP1A, MEP1B, and RUVBL2 (Reptin), respectively) and the ABI BioMark HD reader (Fluidigm). Expression data (Ct values) were acquired using Fluidigm Real-Time PCR Analysis software (4.1.3). Gene expression was normalized with the RNA ribosomal 18 S, and the level of expression of the tumor sample was compared with the mean level of the corresponding gene expression in normal liver tissues, expressed as an n-fold ratio. The relative amount of RNA was calculated with the 2^−ΔΔCt^ method.

For cell culture samples, real time quantitative PCR was performed using the StepOnePlus^TM^ Real-Time PCR System (Applied Biosystems) with B-R SYBR^®^ Green SuperMix for iQ^TM^ (Quanta Biosciences). Primers used are shown on Table 2. The threshold cycle (C^t^) value for each gene was normalized to the C^t^ value for RNA18S and all relative levels of expression (2^-ΔΔCt^) were calculated [41]. All samples were analyzed at least in triplicate.

**Table 2:** PCR primers

Gene	Forward	Reverse
MEP1A	5'-ATTTCAACAGTTTGATGGGTGCT-3'	5'-ATGGCCTTATAGGCACATCCT-3'
MEP1B	5'-AACACGGTGCCCTCATCATA-3'	5'-CCTGCATTAGTCACATGGGC-3'

### Western blot

Cells were lysed in a 1% SDS PBS solution supplemented with protease inhibitor cocktail (Roche). Western blot was done as described previously [3]. All blots were analyzed with the Odyssey^®^ system (Li-Cor Biosciences). The following antibodies were used: Reptin (Clone 2E9-5 Sigma-Aldrich), meprin α (Human Meprin α Subunit/MEP1A Antibody R&D Systems), meprin ß (Anti-ß/mMEP1B R&D Systems), tubulin (mouse monoclonal, Sigma-Aldrich).

### Immunohistochemistry

Tissues were obtained from patients with HCC undergoing surgical resection or liver transplantation. Patients gave informed consent and procedures followed the French regulations. Formalin fixed paraffin-embedded sections were deparaffinized and incubated overnight at 56°C in citrate buffer, pH 9 in a PT LINK (Dako). Following inhibition of endogenous peroxidases, slides were incubated with a rabbit antibody against human meprin α (home-made against a peptide located at the molecule surface of meprin α, Pineda Antibody-Service), then with Dako Flex reagents.

### Statistics

Statistical analyses were performed using the GraphPad Prism 6.0 software.

The project was funded by a grant from Ligue Nationale Contre le Cancer (Equipe Labélisée 2011) (to JR) and by the Deutsche Forschungsgemeinschaft (DFG) SFB877 “Proteolysis as a Regulatory Event in Pathophysiology” (projects A9) and grant BE4086/5-1 (to CBP). U1162 team is funded by the Ligue Nationale Contre le Cancer (Equipe Labelisée 2014) and the Labex OncoImmunology.

## SUPPLEMENTARY MATERIALS FIGURES


