# Supplementary material for: Metalloproteinase meprin α regulates migration and invasion of human hepatocarcinoma cells and is a mediator of the oncoprotein Reptin
Source: Oncotarget. 2016 Dec 16;8(5):7839–51. doi: 10.18632/oncotarget.13975 (PMC5352365; doi:10.18632/oncotarget.13975)
Supplement: Supplementary file 1 [file oncotarget-08-7839-s001.pdf]

Metalloproteinase meprin α regulates migration and invasion of human hepatocarcinoma cells and is a mediator of the oncoprotein Reptin

Supplementary Materials

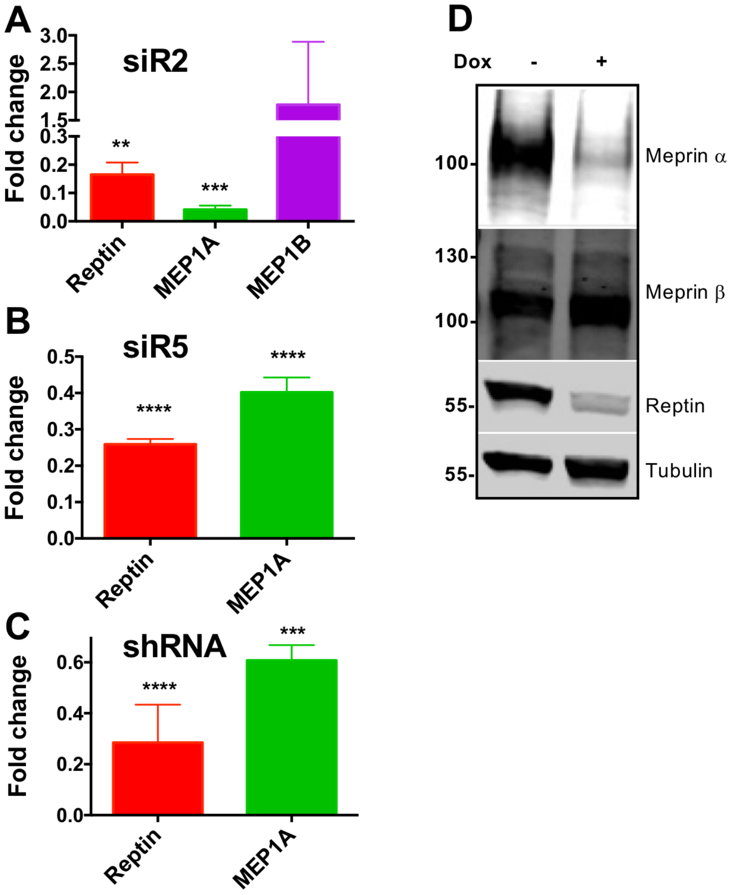

Supplementary Figure S1: Reptin regulates expression of meprin α in Hep3B cells. (**A**) Expression of Reptin, meprin α and meprin ß mRNA was examined by RT-PCR in HuH7 cells following Reptin silencing with the siR2 siRNA. Results are expressed as fold changes as compared to treatment with a control siRNA ($p < 0.0001$; $n = 5$ for Reptin, 8 for MEP1A and 4 for MEP1B). (**B**) Same experiment with the siR5 siRNA ($p < 0.0001$; $n = 3$). (**C**) Same experiment but Reptin was silenced with a doxycycline-inducible shRNA. Results are expressed as fold changes as compared to non-induced cells ($p < 0.0001$; $n = 3$ for Reptin and $n = 4$ for MEP1A). (**D**) Western blot of Hep3B extracts showing expression of meprin α, ß, and Reptin following silencing Reptin with the inducible Reptin shRNA. Tubulin is showed as a loading control.

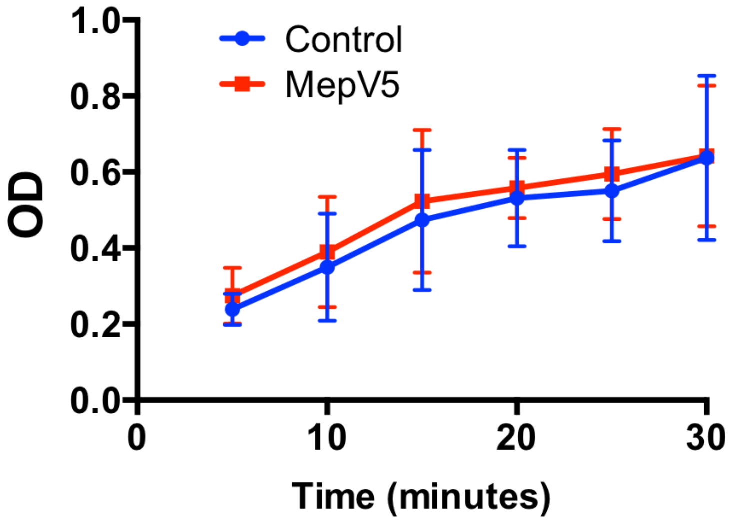

Supplementary Figure S2: Overexpression of meprin α does not alter adhesion of cells. Control HuH7 cells or cells stably overexpressing meprin α were seeded on serum-coated wells. At indicated times, the cells were washed and the number of adherent cells was estimated with the MTS assay. The graph shows the mean of 3 experiments conducted in 6 replicates/data point. There was no significant difference in adhesion between the two cell types.
